# Numerical Simulation on Size Effect of Fracture Toughness of Concrete Based on Mesomechanics

**DOI:** 10.3390/ma13061370

**Published:** 2020-03-18

**Authors:** Juan Wang, Qianqian Wu, Junfeng Guan, Peng Zhang, Hongyuan Fang, Shaowei Hu

**Affiliations:** 1 School of Water Conservancy Engineering, Zhengzhou University, Zhengzhou 450001, China; wangjuan@zzu.edu.cn (J.W.); wqq1719@163.com (Q.W.); 18337192244@163.com (H.F.); hushaowei@nhri.cn (S.H.); 2 School of Civil Engineering and Communication, North China University of Water Resources and Electric Power, Zhengzhou 450045, China; shuaipipi88@126.com; 3 College of Civil Engineering, Chongqing University, Chongqing 400045, China

**Keywords:** concrete, size effect, fracture toughness, tensile strength, initial defects

## Abstract

The fracture performance of concrete is size-dependent within a certain size range. A four-phase composite material numerical model of mesofracture considering a mortar matrix, coarse aggregates, an interfacial transition zone (ITZ) at the meso level and the initial defects of concrete was established. The initial defects were assumed to be distributed randomly in the ITZ of concrete. The numerical model of concrete mesofracture was established to simulate the fracture process of wedge splitting (WS) concrete specimens with widths of 200–2000 mm and three-point bending (3-p-b) concrete specimens with heights of 200–800 mm. The fracture process of concrete was simulated, and the peak load (*P*_max_) of concrete was predicted using the numerical model. Based on the simulating results, the influence of specimen size of WS and 3-p-b tests on the fracture parameters was analyzed. It was demonstrated that when the specimen size was large enough, the fracture toughness (*K*_IC_) value obtained by the linear elastic fracture mechanics formula was independent of the specimen size. Meanwhile, the improved boundary effect model (BEM) was employed to study the tensile strength (*f*_t_) and fracture toughness of concrete using the mesofracture numerical model. A discrete value of *β* = 1.0–1.4 was a sufficient approximation to determine the *f*_t_ and *K*_IC_ values of concrete.

## 1. Introduction

The mechanical behavior and fracture properties of engineering materials are significantly dimensionally dependent. As early as the 17th century, Mariotte [1] observed from strength tests of different materials that the load-bearing capacity was affected by specimen size because of the heterogeneity of materials. Griffith [2] discovered the size effects of fracture parameters of engineering materials through experiments and interpreted it as the effects of defects. A large number of results have also shown that the strength and fracture parameters of concrete obtained from experiments vary with the size of specimens [3,4,5,6,7]. Exploration of the regularity and mechanism of the size effect phenomenon has never stopped. However, by comparing a series of experimental results of specimens with various sizes, it is concluded that the size effect phenomenon exists at a certain scale range. For example, Wittmann [3] tested compact tensile specimens with section heights of 360, 720 and 1440 mm. It was concluded that the fracture energy of concrete is independent of the specimen’s size when the ligament length reaches approximately 20 times the maximum aggregate diameter. It was also considered that when the height of the specimen reaches 800 mm, the measured fracture toughness *K*_IC_ value no longer has size effects [4]. The similar conclusion drawn by Bazant [5] is that the mechanical parameters measured for specimens in a certain size range had size effects. That is to say, when the size of the specimens was small enough or large enough, the mechanical properties and fracture parameters remained unchanged. In view of this experimental phenomenon, Weibull [8,9] considered that the size dependence was due to the increase in the probability of encountering low-strength material elements with increasing structure sizes. Based on the law of extreme strength distribution, a size effect statistical theory was proposed. However, this theory was limited to structures that fail at the beginning of macro-cracks and small structures that only cause negligible stress redistribution in the fracture process zone when they fail. Subsequently, based on the energy theory, Bazant et al. [5] proposed a size effect theory of fracture mechanics for geometrically similar specimens with a size range of approximately 1:20 notches. Based on the concept of fractals, Carpinteri et al. [6] established a multi-fractal size effect law that reflected the unstable cracking of structures when the size range was approximately 1:10. Hu [7] concluded that the initial crack and ligament depth should be far from the specimen boundary to reflect the true mechanical parameters of a material, independent of size. Based on this, the boundary effect theory was established. In addition to this basic theory, indirect size effects such as the boundary layer effect [10], time-dependent size effects caused by diffusion phenomenon [11], and time-dependent size effects with respect to a material’s constitutive relationship have also been included [12]. Based on fractal theory, Huang et al. [13] established a fractal model for the size effect of the fracture energy of concrete. Huang [14] analyzed the existing theory of strength size laws and the phenomenon of size effects of fracture parameters and determined the strength size effects of different types of concrete and a research method for small size specimens with a brittleness index. With developments concerning the size effect model and boundary effect model [15,16,17] in recent years, the mechanism and scale law of concrete strength have become clearer.

Although there is not an agreed-upon explanation for the influence of sample size on concrete strength, its existence was approved and was considered to be large enough [3,4]. Considering the limitations of the objective conditions of the laboratory, it is difficult to carry out ultra-small size and ultra-large size tests, and the corresponding results are relatively rare. Therefore, it is necessary to use numerical simulation technology to study the fracture behavior of large-scale concrete. In fact, mesoscale numerical models have been used to research the mechanical behavior of concrete to reveal the strength mechanism [18,19] and have been verified to be applicable to the study of the fracture process and the influences of microstructures on concrete properties.

In this study, a meso-mechanical fracture model of concrete was established based on the results of concrete microstructure experiments and was used to simulate the fracture process of wedge splitting (WS) specimens with widths of 200–2000 mm and three-point bending (3-p-b) specimens with heights of 200–800 mm of concrete. The fracture parameters and dimension-independent strength of concrete were analyzed using the simulation results.

## 2. Numerical Model and Examples

### 2.1. Generation of Concrete Mesostructure

**Figure 1:**
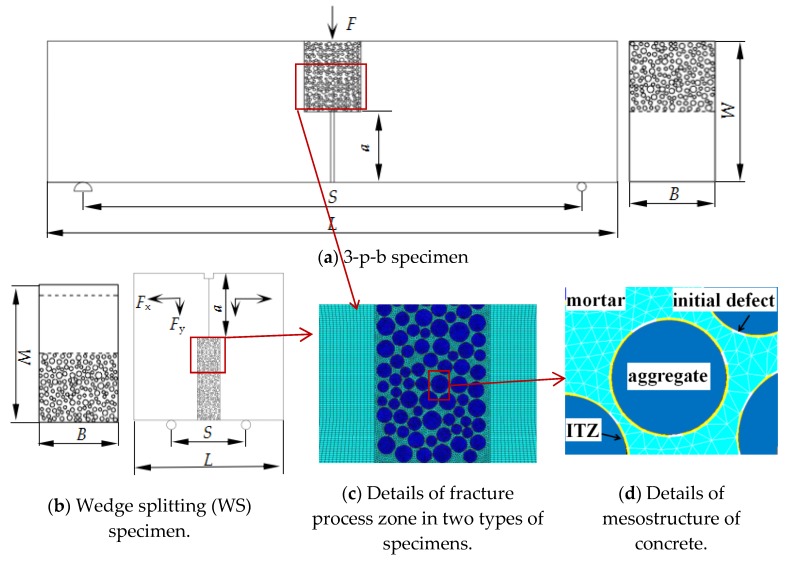
Schematic diagram of concrete meso-model.

As the weak part of concrete, the internal porosity of the interfacial transition zone (ITZ) was much higher than that of a mortar matrix. Concrete was prone to fracture because of initial defects in its interior; stress was concentrated near a defect, and the main crack was formed by defect propagation and convergence, which led to macro cracks or the failure of the concrete [20,21,22]. Therefore, initial defects were necessary to be considered in the mesostructure model of concrete and were assumed to be distributed randomly in the interfacial transition zone (ITZ) of concrete. Thus, concrete was modeled as a four-phase [23,24,25] composite material consisting of a mortar matrix, coarse aggregates, an ITZ and initial defects at the meso level (Figure 1d). WS and 3-p-b concrete specimens were used.

The finite element model of the concrete mesostructure was generated and fracture process was simulated by using ANSYS (14.0, PA, USA). The model parameters were determined according to the experiments given in [26], in which the coarse aggregate volume fraction was 45%, the diameters were 5–10 mm, the diameter gradation was determined by the Fuller curve and Walraven formula [27], and the locations were generated by the Monte Carlo method. Solid elements were adopted in the finite element model. Two-dimensional (2D) four-node solid elements with two degrees of freedom at each node were used. The initial defects were simulated by the solid elements with weakened properties, and the content of the initial defect was defined in Equation (1). The depth of the ITZ varied from 0.02 to 0.2 mm according to the research results [28,29,30,31,32]. The influence of ITZ depth on the simulation results of concrete strength was studied in [33] and drew the conclusion that the depth of ITZ had little influence on the failure mode and strength of concrete. Therefore, the depth of ITZ was defined to be 0.2 mm considering the simulation efficiency and research results. The mesostructure of the four-phase material model was only applied to the fracture process zone with a width of 60 mm (6 *d*_max_), as shown in Figure 1a,b.

(1) $$p=N_{1}/N_{2}$$

where *p* is the initial defect content, *N*_1_ is the number of ITZ defect units and *N*_2_ is the total number of ITZ units.

To study the effects of size on concrete fracture performance, specimens with initial notch (*a*) were generated, which sizes varying from 200 to 2000 mm. Specifically, the sizes of 3-p-b specimens (*W* × *S*) were 200 × 800 mm, 300 × 1200 mm, 400 × 1600 mm, 500 × 2000 mm, 600 × 2400 mm, and 800 × 3200 mm, with thicknesses (*B*) of 200 mm. The sizes of WS specimens (*W* × *L*) were 200 × 200 mm, 400 × 400 mm, 600 × 600 mm, 800 × 800 mm, 1000 × 1000 mm, 1200 × 1200 mm, 1500 × 1500 mm, and 2000 × 2000 mm, with thicknesses (*B*) of 200 mm. A displacement load and constraints were imposed on the 3-p-b and WS specimens, according to experiments, as shown in Figure 1a,b. It must be specially noted that for WS specimens, the displacement loads *F*_x_ and *F*_y_ applied in the *X*, *Y* directions were calculated by Equation (2).

(2) $$F_{y}/F_{x}=\tan15^{\circ }$$

### 2.2. Constitutive Relations and Failure Criteria

**Figure 2:**
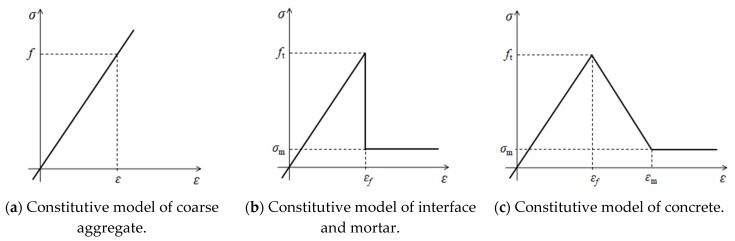
Constitutive relationships among components.

At the meso-level, the coarse aggregates were regarded as a linear elastic material and a linear elastic constitutive model was adopted, as shown in Figure 2a. An elastic–brittle constitutive model was used for mortar and ITZ, as shown in Figure 2b. A nonlinear damage constitutive model (Equation (3)) was used for concrete, as shown in Figure 2c. The maximum principal stress failure criterion was used, i.e., when the maximum principal stress of the element was greater than its allowed strength, the element began to fail.

(3) $$\sigma =\left\{ \begin{array}{cc} E\varepsilon & 0\leq \varepsilon \leq \varepsilon_{f}\\ f_{t}\left[1-{\left(\frac{\varepsilon -\varepsilon_{f}}{\varepsilon_{m}-\varepsilon_{f}}\right)}^{n}\right] & \varepsilon_{f}\leq \varepsilon \leq \varepsilon_{m}\\ \sigma_{m} & \varepsilon_{m}\leq \varepsilon \end{array}\right.$$

where *ε* is the principal strain of concrete, *E* is the elastic modulus, *σ* is the principal stress, *ε_f_* is the peak strain, *ε*_m_ is the ultimate strain, *σ*_m_ is the residual strength, *f_t_* is the tensile strength and *n* is the softening coefficient, assigned a value of one.

### 2.3. Determination of Property Parameters

The mechanical properties of the primary materials are listed in Table 1. Among them, the tensile strength and elastic modulus of mortar were calculated according to empirical formulas shown in Equations (4)–(6), as described in [34,35]. The value of *c*/*w* was determined according to [26]. Considering there were difficulties in developing mechanical experiments regarding the ITZ, its properties were determined based on the research results of existing references [36].

(4) $$E_{m}=1000\left(7.7\ln\left(f_{\mathrm{cm}}\right)-5.5\right)$$

(5) $$f_{\mathrm{tp}}=1.4\ln\left(f_{\mathrm{cm}}\right)-1.5$$

(6) $$c/w=0.047f_{\mathrm{cm}}+0.5$$

where *E*_m_ is the elastic modulus of mortar, *f*_tp_ is the pure tensile strength of mortar, *f*_cm_ is the compressive strength of mortar and *c*/*w* is the cement–water ratio of mortar.

### 2.4. Determination of Mesh Size

**Figure 3:**
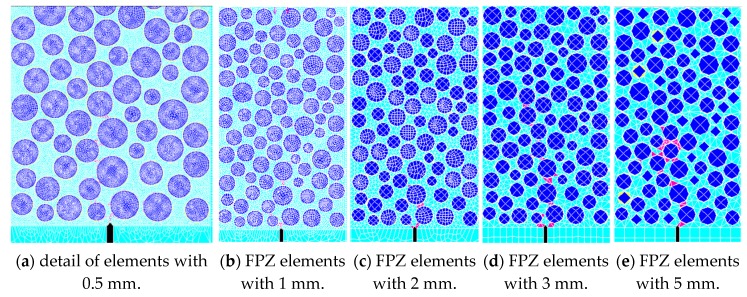
Diagram of cracks in fracture process zone (FPZ) of three-point bending (3-p-b) specimens with different mesh sizes (blue elements for coarse aggregates and red color for failure elements in interfacial transition zone (ITZ) and mortar).

To explore the influence of element size on the simulation results of the numerical model, we analyzed the results of concrete fracture when the mesh size of the mortar and the interface were at sizes of 0.5, 1, 2, 3 and 5 mm respectively. The results were shown in Table 2 and Figure 3. It can be seen that the failure mode is not dependent on mesh size, while the peak load *P*_max_ changes when mesh size varies. Combined with the results of the experiment in [26], when the mesh size was in the range from 0.5 to 2 mm, the simulating results were relatively stable and reasonable. In this paper, the mesh size of the meso structure was 1 mm, and then it gradually increased to 5 mm in the macro structure.

## 3. Results and Discussion

### 3.1. Cracking Process

**Figure 4:**
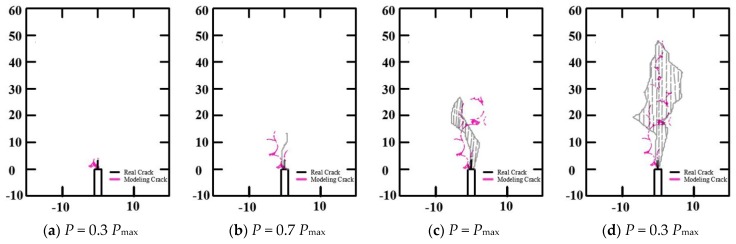
Evolution process of the 3-p-b specimen fracture process zone.

**Figure 5:**
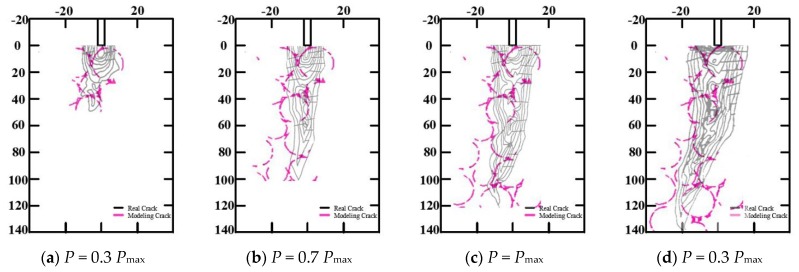
Evolution process of the WS specimen fracture process zone.

The fracture processes of concrete 3-p-b specimens and WS specimens were simulated and compared with the experimental results of [37], as shown in Figure 4 and Figure 5, respectively. There were no obvious differences between the fracture phenomena of the 3-p-b specimens and WS specimens of concrete for the final failure mode. When the load reached 30% of *P*_max_, the concrete specimen of 3-p-b cracked from the initial notch tip, and when the load reached 17% of *P*_max_, the concrete specimen of WS cracked from the initial notch tip—thereby, the first microcracking or strain localization occurred and the inner microcracks accumulated [38] in both types. As the load continued to increase, cracks gradually expanded and converged with the crack at the crack tip, forming a macroscopic visible main crack at the crack tip in the fracture process zone, as shown in Figure 4b and Figure 5b [39]. Subsequently, the cracks steadily expanded as the external load gradually increases to the peak load. Then, cracks expanded unsteadily and the load began to decrease, ultimately causing the concrete fracture failure with increasing load. It could be concluded that the primary crack of WS and 3-p-b specimens propagates along the surface of coarse aggregates, so the shape, distribution, and particle size of coarse aggregates played important roles in the concrete fracture process [40]. These fracture process simulation results agreed well with the experimental results of [37,41].

### 3.2. Peak Stress

**Figure 6:**
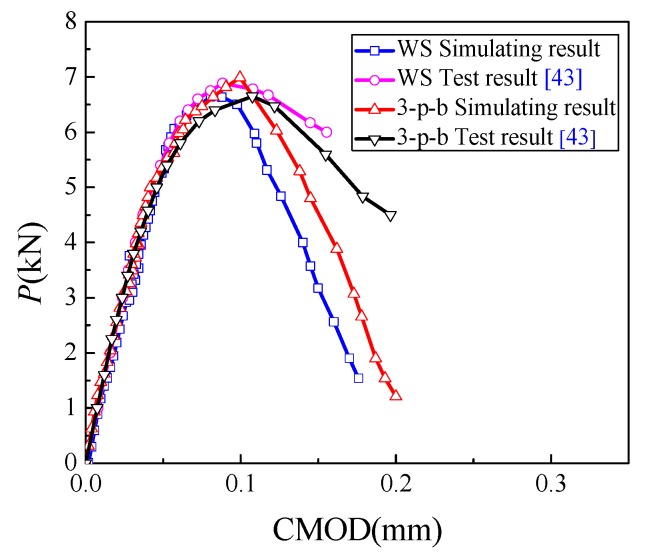
*P*-CMOD (Crack Mouth Opening Displacement) curves of WS and 3-p-b specimens [43].

The fracture toughness and tensile strength of concrete depend on the peak load. The meso-mechanical fracture model generated in Section 2 was used to simulate the concrete fracture tests of WS and 3-p-b specimens, and proportional ultimate strength *f*_L_ was calculated accordingly by Equation (7) [42]. The Load-Crack Mouth Opening Displacement (*P*-CMOD) curves of numerical simulation results and test results of WS and 3-p-b specimens with a dimension of 200 mm in height are shown in Figure 6. Here, the values of specimen parameters, such as the ratio of the initial notch to the height of the specimen (*a*/*W*), specimen height (*W*), and specimen thickness (*B*) were calculated according to the numerical model, which was determined using Xu’s experimental data [26]. From the results presented in Figure 6 and Table 3 and Table 4, it can be seen that the simulation results are more brittle. That may be due to the elastic constitutive relation of meso materials. However, the simulation results have good discreteness and agree well with the experimental results before the peak point. To analyze the influence of specimen size on proportional ultimate strength and find the independent size of concrete strength, the fracture process of concrete specimens with heights larger than 1200 mm was further simulated, as shown in Table 3 and Table 4.

(7) $$f_{L}=\frac{3P_{\max}S}{2Bh^{2}}$$

where *h* is the effective height of the specimen section, *h* = *W* − *a*.

The failure mode and crack propagation process did not significantly change with an increase in the specimen’s dimension; meanwhile, the proportional ultimate strength *f*_L_ and the crack length decreased with increasing concrete specimen dimensions for specimens of both types. However, the growth of 3-p-b specimens was slower than that of WS specimens.

### 3.3. Fracture Toughness

**Figure 7:**
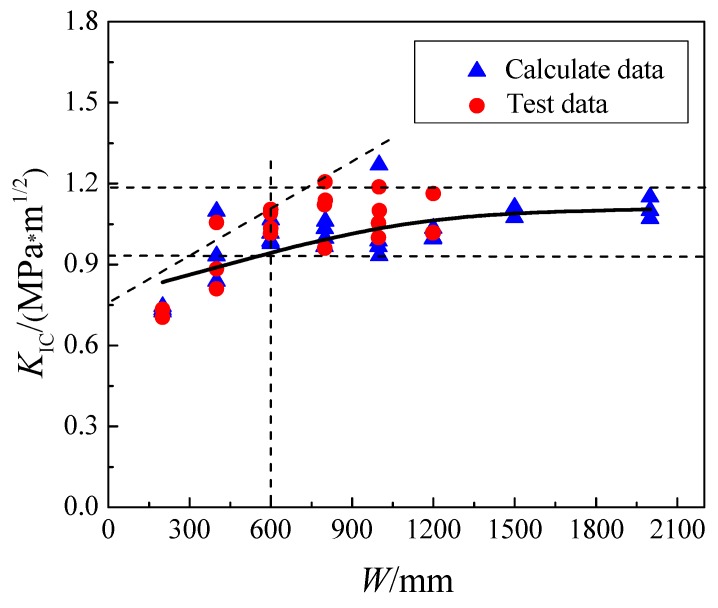
Effects of specimen height on fracture toughness *K*_IC_.

It has been concluded [4] that the fracture toughness *K*_IC_ of concrete increases with increasing of specimen size, and when the specimen size is large enough, the change in *K*_IC_ with size is not noticeable [44,45,46]. To verify that *K*_IC_ varies with size, as well as to find the scale-independent fracture parameters of concrete, the above experimental results and numerical simulation results were substituted into Equation (8) [47,48], and the fracture toughness of concrete was calculated and is shown in Table 5. According to the results, the variation of fracture toughness *K*_IC_ with specimen height *W* was drawn and is shown in Figure 7. It can be concluded that the fracture toughness increases with the increase in specimen size. When the height of the specimen reaches 600 mm (*W*/*d*_max_ = 60), the trend line is basically completely within the dashed lines, the fracture toughness no longer changes significantly and is approximately 1.10 MPa·m^1/2^, independent of the size of the specimen.

(8) $$K_{I}B\sqrt{W}/2P=3.675{\left[1-\frac{a}{W}\right]}^{-3/2}$$

where *P*_max_ is the peak load, *W* is the beam height, *B* is the beam thickness and *a* is the initial notch length.

### 3.4. Discussion of the Size Effect on Fracture Toughness and Tensile Strength

It is agreed that the fracture toughness will not increase when the specimen’s size is large enough [49,50]. Compared with the other classic size effect law [5,6,8,9], the boundary effect model [51,52,53,54,55] needs less input data (only peak load *P*_max_) to determine the size-independent tensile strength *f*_t_ and fracture toughness *K*_IC_, shown as Equation (9), which is a more suitable application for the numerical model. The peak loads (*P*_max_) obtained from the above numerical model, the experiments of [30] and the parameters involved can be used with this equation to determine the tensile strength of concrete specimens.

(9) $$\frac{1}{\sigma_{n}^{2}\left(P\right)}=\frac{1}{f_{t}^{2}}+\frac{1}{f_{t}^{2}}\frac{a_{e}}{a_{\infty }^{*}}=\frac{1}{f_{t}^{2}}+\frac{4a_{e}}{K_{IC}^{2}}$$

where $a_{\infty }^{*}$ is the characteristic crack length of material, fully determined by *f*_t_ and *K*_IC_. *a*_e_ is the equivalent crack length, fully determined by the specimen size, type and *a*, which can be described as *a*_e_ = *B*(*α*)*a*, where *B*(*α*) is the geometric shape parameter for 3-p-b specimens [15,22,23,56,57].

(10) $$B\left(\alpha \right)={\left[\frac{A\left(\alpha \right)Y\left(\alpha \right)}{1.12}\right]}^{2}$$

(11) $$A(a)={\left(1-a\right)}^{2}$$

(12) $$Y\left(\alpha \right)=\frac{1.99-\alpha \left(1-\alpha \right)\left(2.15-3.93\alpha +2.7\alpha^{2}\right)}{\sqrt{\prod }\left(1+2\alpha \right){\left(1-\alpha \right)}^{3/2}}$$

Concrete is a highly heterogeneous composite material. The evolution of the concrete micro-fracture process zone shows that the distribution of coarse aggregates and particle size plays important roles in the crack growth. According to [15,22,23,56,57], the virtual crack length at the tip of the initial notch (Δ*a*) at *P*_max_ depends on the maximum size of coarse aggregates, and a discrete coefficient (*β*) can be introduced to describe the relationship between Δ*a* and the maximum diameter of coarse aggregates (*d*_max_), shown as Equation (13).

(13) $$\Delta \alpha =\beta d_{\max}$$

For different types of concrete specimens, the value of *β* can be customized. Therefore, the nominal stress can be expressed as Equation (14).

(14) $$\sigma_{n}\left(P_{\max}\right)=\frac{\frac{S}{B}P_{\max}}{\frac{W_{1}W_{2}^{3}+W_{1}^{4}+6\Delta aW_{1}^{2}W_{3}}{3W_{3}^{2}}+2{\left(\Delta a\right)}^{2}}$$

where,

(15) $$W_{1}=W-a-\Delta a$$

(16) $$W_{2}=W-a+\Delta a$$

(17) $$W_{3}=W-a$$

**Figure 8:**
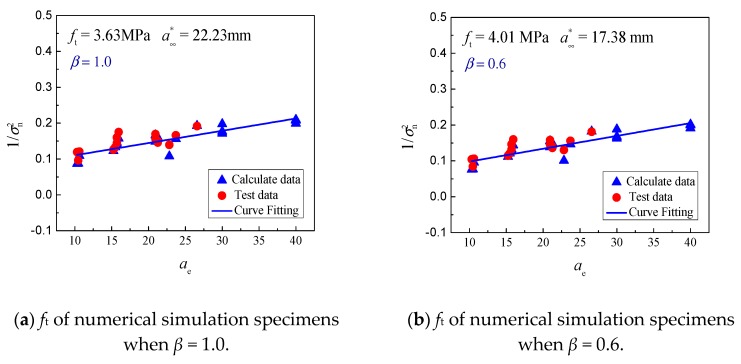

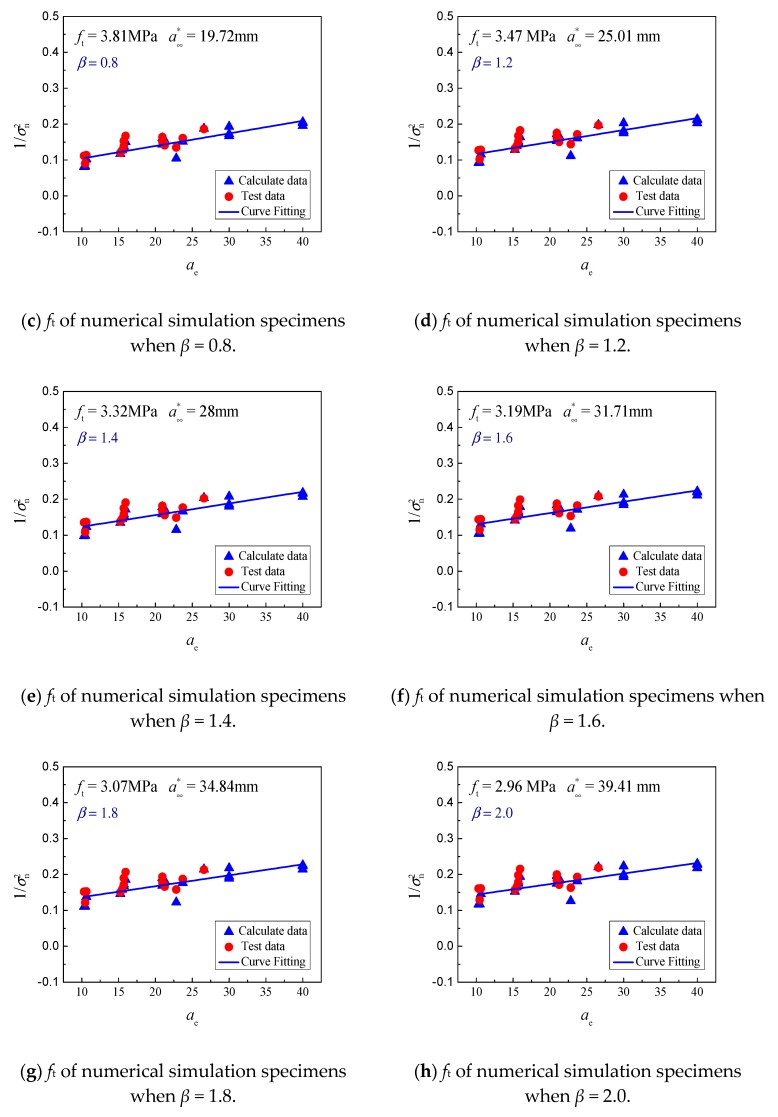

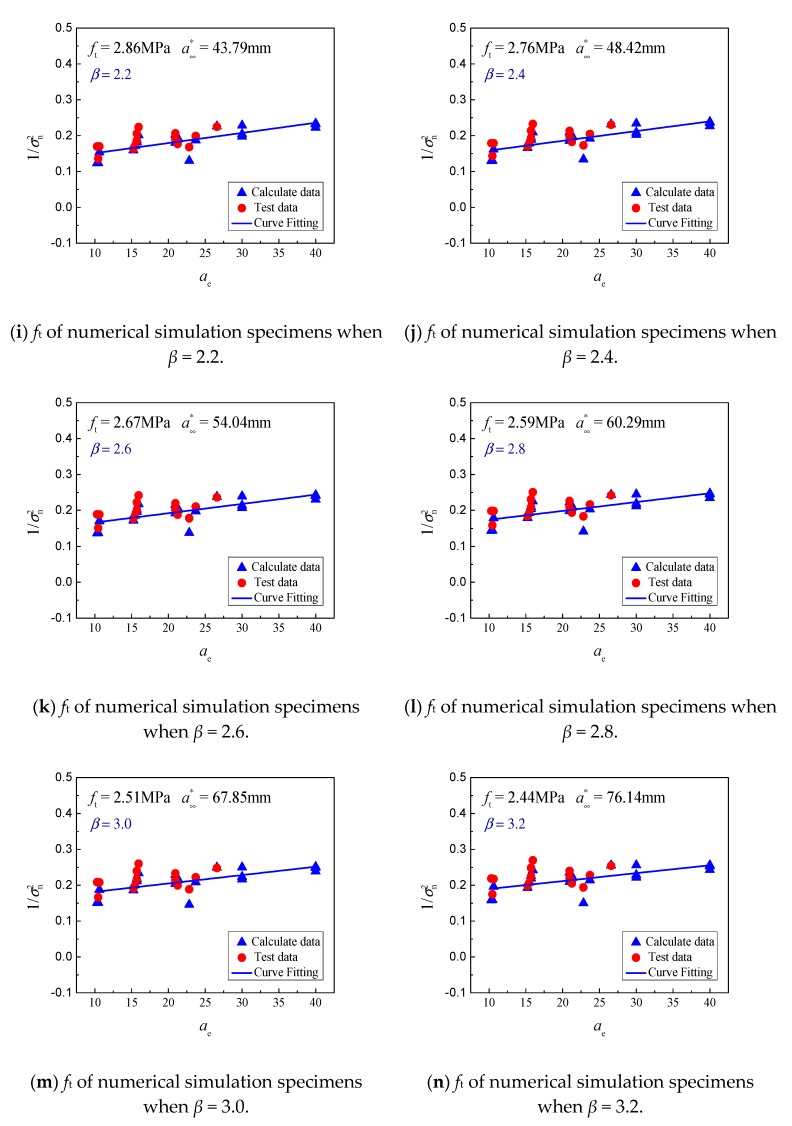
Tensile strength (*f*_t_) of simultaneous numerical simulation specimens with various values of *β.*

Accordingly, the fracture toughness *K*_IC_ and tensile strength *f*_t_ independent of size can be regressed by measuring the peak load *P*_max_ of various size specimens. The fitting results of tensile strength and fracture toughness under various conditions of ∆*a* are shown in Figure 8. When *β* ranges from 0.6 to 3.2, the tensile strength (*f*_t_) and fracture toughness (*K*_IC_) vary from 2.44 to 4.01 MPa and 1.06 to 1.35 MPa·m^1/2^, respectively. It is known that the ratio of concrete tensile strength to compressive strength is approximately 1/8–1/12, and the compressive strength in [26] was 29.56 MPa. It can, thus, be inferred that the value of *f*_t_ varies from 2.46 to 3.70 MPa. Meanwhile, the fracture toughness of concrete (*K*_IC_) is relatively stable at approximately 1.10 MPa·m^1/2^. It can be concluded that the value of *β* is 1.0–1.4, and reasonable tensile strength *f*_t_ and fracture toughness *K*_IC_ can be obtained for small aggregate fracture specimens. The results agree well with those of [53], which were reached based on concrete fracture experiments with various specimen types. In conclusion, the model can be used to simulate the fracture tests of small size concrete under different conditions. Based on this, the fracture toughness and tensile strength, independent of size, can be further determined by the boundary effect theory.

## 4. Conclusions

To analyze the influence of specimen size on concrete fracture parameters, a meso-mechanical fracture model of concrete considering initial defects was established, and fracture tests of concrete WS specimens and 3-p-b specimens were simulated accordingly. The tensile strength and fracture toughness were determined by the boundary effect theory based on the numerical simulation results of concrete fracture tests. The main conclusions are given as follows.

(1) The numerical model of concrete mesofracture, considering initial defects, can simulate the fracture process and predict the peak load of concrete, so it is suitable for determining concrete fracture parameters and tensile strength;
(2) Based on the above mesofracture numerical model, when the height of a concrete specimen reaches 600 mm (*W*/*d*_max_ = 60), the fracture toughness *K*_IC_ calculated from *P*_max_ and the initial notch length according to the linear elastic fracture mechanics formula is independent of the specimen size;
(3) The tensile strength (*f*_t_) and the fracture toughness (*K*_IC_) which are independent in specimens of concrete can be obtained by the application of the mesofracture numerical model and the BEM. This property can be well expressed by ∆*a* at peak load (*P*_max_), and the relationship between ∆*a* and the maximum aggregate diameter (*d*_max_) can be established by introduced a discrete coefficient (*β*). Discrete values of *β* range from 1.0 to 1.4 are a sufficient approximation to predict the *f*_t_ and *K*_IC_ values of concrete.

## Figures and Tables

**Table 1:** Properties of primary materials.

Microscopic Components	Elastic Modulus (GPa)	Strength (MPa)	Poisson’s Ratio	Volume Content (%)
Aggregate	60	—	0.168	45
Mortar	18	2.5	0.2	—
Interface	5.4	0.83	0.2	—
Concrete	20	1.0	0.168	—
Interface initial defects	5 × 10^−6^	—	—	30

**Table 2:** Mesh size sensitivity analysis of concrete meso numerical model.

Mesh Size (mm)	Element Number	Simulation Results *P*_max_ (kN)	Relative Error of *P*_max_ /%
0.5	67458	6.58	4.1
1	19403	6.84	0.2
2	8530	7.02	2.3
3	5467	9.08	32.3
5	4119	10.24	49.3

**Table 3:** Simulation results for WS specimens.

Specimen Number	*a*/*W*	*W* (mm)	*B* (mm)	Test Result *f*_L_ (MPa)	Simulation Results *f*_L_ (MPa)	Relative Error (%)
I-W200-1	0.46	200	200	0.91	0.94	0.0
I-W200-2	0.47	200	200	0.92	0.89	
I-W200-3	0.47	200	200	0.88	0.88	
Average				0.90	0.90	
I-W400-1	0.45	400	200	0.70	0.73	5.0
I-W400-2	0.46	400	198	0.93	0.82	
I-W400-3	0.46	400	199	0.78	0.96	
Average				0.80	0.84	
I-W600-1	0.46	600	193	0.79	0.76	3.9
I-W600-2	0.46	599	200	0.73	0.71	
I-W600-3	0.46	600	193	0.74	0.70	
I-W600-5	0.46	599	200	0.78	0.73	
Average				0.76	0.73	
I-W800-1	0.45	799	196	0.59	0.64	8.7
I-W800-2	0.46	800	194	0.75	0.62	
I-W800-4	0.46	798	200	0.70	0.60	
I-W800-5	0.46	801	200	0.71	0.66	
Average				0.69	0.63	
I-W1000-1	0.45	997	200	0.55	0.53	5.0
I-W1000-3	0.45	997	200	0.58	0.54	
I-W1000-4	0.45	999	196	0.65	0.70	
I-W1000-5	0.45	1000	200	0.60	0.51	
Average				0.60	0.57	
I-W1200-0	0.45	1198	200	0.51	0.50	5.6
I-W1200-1	0.46	1200	201	0.52	0.52	
I-W1200-2	0.45	1200	200	0.58	0.50	
Average				0.54	0.51	
I-W1500-1	0.5	1500	200	—	0.44	—
I-W1500-2	0.5	1500	200	—	0.44	
I-W1500-3	0.5	1500	200	—	0.43	
Average					0.44	
I-W2000-1	0.5	2000	200	—	0.40	—
I-W2000-2	0.5	2000	200	—	0.37	
I-W2000-3	0.5	2000	200	—	0.38	
Average					0.38	

**Table 4:** Numerical simulation results for 3-p-b specimens.

Specimen Number	*a*/*W*	*W* (mm)	*B* (mm)	Test Result *f*_L_ (MPa)	Simulation Results *f*_L_ (MPa)	Relative Error (%)
II-T200-2	0.47	200	200	3.84	4.03	8.9
II-T200-3	0.48	200	200	3.46	4.06	
II-T200-4	0.46	199	199	3.47	3.65	
Average				3.59	3.91	
II-T300-1	0.47	298	200	2.85	2.99	2.7
II-T300-2	0.47	297	195	3.10	3.14	
II-T300-3	0.46	298	198	2.75	2.90	
II-T300-4	0.48	298	200	3.14	3.14	
II-T300-5	0.49	298	200	3.23	3.27	
Average				3.01	3.09	
II-T400-1	0.46	401	199	2.86	2.76	0.7
II-T400-2	0.46	400	196	2.88	2.79	
II-T400-3	0.47	396	199	2.84	2.92	
II-T400-4	0.47	396	197	2.75	2.78	
Average				2.83	2.81	
II-T500-1	0.53	499	200	2.68	2.76	5.6
II-T500-2	0.46	500	196	2.50	2.50	
II-T500-3	0.55	500	198	2.39	2.72	
Average				2.52	2.66	
II-T600-1	0.5	600	200	—	2.40	—
II-T600-2	0.5	600	200	—	2.54	
II-T600-3	0.5	600	200	—	2.58	
Average					2.51	
II-T800-1	0.5	800	200	—	2.36	—
II-T800-2	0.5	800	200	—	2.30	
II-T800-3	0.5	800	200	—	2.31	
Average					2.32	

**Table 5:** Fracture toughness (*K*_IC_) calculation results.

Specimen Number	*K*_IC_/(MPa·m^1/2^)	Average	Test *K*_IC_/(MPa·m^1/2^)	Test Average
I-W200-1	0.7238	0.7329	0.7331	0.7236
I-W200-2	0.7433		0.7337	
I-W200-3	0.7316		0.7039	
I-W400-1	0.8376	0.9553	0.8099	0.9161
I-W400-2	0.9318		1.0553	
I-W400-3	1.0964		0.883	
I-W600-1	1.0655	1.0111	1.1039	1.0607
I-W600-2	0.9860		1.0153	
I-W600-3	0.9776		1.0333	
I-W600-5	1.0153		1.0901	
I-W800-1	1.0326	1.0140	0.9596	1.1062
I-W800-2	0.9965		1.2062	
I-W800-4	0.9662		1.1212	
I-W800-5	1.0607		1.1377	
I-W1000-1	0.9655	1.0383	0.9993	1.0846
I-W1000-3	0.9862		1.053	
I-W1000-4	1.2689		1.187	
I-W1000-5	0.9325		1.0992	
I-W1200-0	0.9948	1.0085	1.0166	1.0657
I-W1200-1	1.0334		1.0183	
I-W1200-2	0.9973		1.1622	
I-W1500-1	1.1118	1.0984	—	—
I-W1500-2	1.1097		—	
I-W1500-3	1.0734		—	
I-W2000-1	1.1495	1.1065	—	—
I-W2000-2	1.0702		—	
I-W2000-3	1.0999		—	
